# Assessment of the Association Between Anthropometric Indices Related to Overweight and Obesity and Selected Trace Elements and Heavy Metals: A Cross-Sectional Study

**DOI:** 10.3390/nu17193141

**Published:** 2025-09-30

**Authors:** Grzegorz Józef Nowicki, Anna Maria Cybulska, Maciej Polak, Elżbieta Grochans, Romuald Bohatyrewicz, Eliza Blicharska, Tomasz Czernecki, Agnieszka Adamczuk, Magdalena Łapot, Barbara Ślusarska

**Affiliations:** 1 Department of Family and Geriatric Nursing, Faculty of Health Sciences, Medical University of Lublin, Staszica 6 Str., 20-059 Lublin, Poland; gnowicki84@gmail.com (G.J.N.); barbara.slusarska@umlub.pl (B.Ś.); 2 Department of Nursing, Faculty of Health Sciences, Pomeranian Medical University in Szczecin, Żołnierska 48 Str., 71-210 Szczecin, Poland; elzbieta.grochans@pum.edu.pl; 3 Department of Epidemiology and Population Studies, Jagiellonian University Medical College, Skawińska 8 Str., 31-066 Krakow, Poland; maciej.1.polak@uj.edu.pl; 4 Department of Anaesthesiology and Intensive Therapy, Faculty of Medicine, Pomeranian Medical University in Szczecin, Unii Lubelskiej 1 Str., 71-252 Szczecin, Poland; romuald.bohatyrewicz@pum.edu.pl; 5 Department of Pathobiochemistry and Interdisciplinary Applications of Ion Chromatography, Medical University of Lublin, Chodźki 1 Str., 20-093 Lublin, Poland; eliza.blicharska@umlub.edu.pl; 6 Department of Biotechnology, Microbiology and Human Nutrition, University of Life Sciences in Lublin, Akademicka 13 Str., 20-950 Lublin, Poland; tomasz.czernecki@gmail.com; 7 Institute of Agrophysics, Polish Academy of Sciences, Doświadczalna 4 Str., 20-290 Lublin, Poland; a.adamczuk@ipan.lublin.pl; 8 Faculty of Medicine, Collegium Medicum, Mazovian Academy in Płock, Pl. Dąbrowskiego 2 Str., 09-402 Płock, Poland; m.lapot@mazowiecka.edu.pl

**Keywords:** trace elements, heavy metals, ferritin, overweight, obesity

## Abstract

**Introduction:** Over the last two decades, obesity has evolved into a global pandemic. Environmental pollutants, as endocrine disruptors, may play a key role in the development of obesity. The study aimed to assess the relationship between the concentration of certain trace elements and heavy metals (Cu, Zn, Mn, Co, Cr and Fe) and ferritin in blood serum, with anthropometric and physiological parameters associated with overweight and obesity in individuals following myocardial infarction and without a previous myocardial infarction. **Method:** The study was conducted in a group of 146 respondents divided into two groups: a study group (SG) of patients that had a history of myocardial infarction (*n* = 74) and a control group (CG) of patients that had no history (*n* = 72). The inductively coupled plasma mass spectrometry was employed to assess the concentration of trace elements and heavy metals. Measurements were taken to determine the anthropometric indices associated with overweight and obesity. **Results:** In the SG, there was a positive correlation between Cr concentration and body adiposity index (BAI) and a negative correlation between Zn, Zn/Cu, and ferritin level and percentage body fat (FM%). In the CG, there was a positive correlation between Zn concentration and WHtR and between ferritin level and BMI, WC, WHR and WHtR. Additionally, a negative correlation was found between Mn concentration and WHR and ferritin level and BAI and FM%. **Conclusions:** This study found a link between certain blood concentrations of trace elements and heavy metals and anthropometric and physiological indices associated with overweight and obesity. It, therefore, has substantial implications for public health.

## 1. Introduction

Since obesity impairs every system in the human body and is considered one of the most prevalent non-communicable diseases (NCDs), over the last 20 years, it has become a serious public health problem. Indeed, it is now considered a global pandemic. According to a 2015 study, 12% of all adults globally—630.7 million—can be considered obese [1]. It is projected that by 2030, 2.16 billion adults globally will be overweight and 1.12 billion adults worldwide will be obese, accounting for nearly 40% of the population.

Obesity is caused by a number of factors, including genetics, energy intake and expenditure imbalances, the consumption of high-calorie foods, sedentary lifestyles, stress and health issues. An increasing amount of research, however, indicates that these factors may not be sufficient to explain obesity, and studies also highlight the growing significance of environmental factors in affecting the development of obesity and the metabolic syndrome [2].

Environmental pollutants, i.e., obesogens, as endocrine disruptors, may have a major impact on obesity development. Obesogens include, among others, phenols, polycyclic aromatic hydrocarbons, amides, metallic compounds, esters, halogenated compounds, air pollutants and flavouring agents. Heavy metals can be found in soil, water, air, dust and the human food chain [3]. It should be noted that heavy metals are a heterogeneous group of elements that can function as both physiological cofactors and toxic elements.

Copper (Cu) is an active, central, structural component of copper/zinc superoxide dismutase (Cu/Zn SOD) that functions to protect cells against superoxide ions. As a result, a moderate amount of copper is essential for lowering oxidative damage and the risk of overweight and obesity [4]. However, excess Cu can disrupt the balance between oxidation and anti-oxidation by catalysing reactive oxygen species (ROS) and reactive nitrogen species (RNS) [5,6]. Zinc (Zn) metabolism disorders induced by Cu displacement lead to increased oxidative stress [7]. Many research investigating the link between serum Cu level and overweight and obesity have produced contradictory results. For example, certain studies have shown that obese people had significantly greater Cu levels than the related control groups [8,9], while other studies have found no correlation between serum Cu level and obesity [10,11]. Gu et al. [12], for example, conducted a meta-analysis assessing the relationship between Cu level and overweight and obesity. Their findings indicate that serum Cu level can be linked to obesity in both children and adults, but the authors recommend more research, taking into account several anthropometric and physiological indices useful in assessing overweight and obesity.

Zinc (Zn) is recognised as one of the key trace elements required for life, playing a critical part in a variety of biochemical and metabolic processes. It plays a catalytic, regulatory and structural role in the energy metabolism of carbohydrates, proteins, and lipids [13]. Zinc (Zn) is a cofactor for over 300 enzymes, including those related to the pathophysiology of obesity [14] and is a structural component of 2500 transcription factors [15,16,17]. A few studies have assessed the link between Zn level and anthropometric parameters connected with overweight and obesity. Some studies have reported considerably lower serum Zn levels in obese individuals than in control groups with normal body weight [8,11,18]; however, other studies have found no significant correlations between serum Zn levels and overweight or obesity [19,20]. In turn, the meta-analysis by Gu et al. [21] found that serum Zn levels were significantly lower in obese children and adults, but the authors emphasised the study’s limitations and the need for additional research.

Manganese (Mn) is a key trace element in the synthesis and activation of numerous enzymes (for instance, oxidoreductases, transferases, hydrolases, lyases, isomerases and ligases), glucose and lipid metabolism, acceleration of protein synthesis, vitamins C and B and catalysis of haematopoiesis, regulation of the endocrine system and enhancement of immune functions [22]. Few studies have investigated Mn levels in overweight or obese people. Still, to the best of the authors’ knowledge, while the mechanism connecting Mn levels to overweight and obesity has not been thoroughly researched, it is recognised that Mn is an antioxidant nutrient that is involved in the production of metalloenzymes and is of key importance in glucose, protein and lipid metabolism [23]. Zhou et al. [24], for instance, discovered that higher manganese intake was connected with a lower risk of abdominal obesity and hypertriglyceridemia in Chinese men. Rotter et al. [25] established that plasma Mn level was considerably higher in obese men aged between 50 and 75 years. Moreover, the American National Health and Nutrition Examination Survey 2011–2014, involving a study group of 5404 children and adolescents aged 6–19, showed that higher blood manganese levels were related to obesity and overweight [26].

Cobalt (Co) is a biologically essential element of vitamin B12 that is involved in the proper functioning of different tissues and organs, as well as DNA production, fatty acid synthesis and energy metabolism [27]. Cobalt (Co) is known to induce hypoxia and is closely associated with oxidative stress and cytotoxicity [28]. Although, past research investigating the link between Co and overweight/obesity is limited, some studies indicate that cobalt exposure plays a role in the development of obesity. Accordingly, Padilla et al. [29] and Wang et al. [30] showed a negative correlation between Co level and body mass index (BMI) and waist circumference (WC). However, these relationships require further verification.

Chromium (Cr) is recognised as an important trace element for humans and its involvement in carbohydrate and fat metabolism is well established [31]. It should be noted that a few studies indicate that Cr may have positive effects in patients with obesity and diabetes [32]. Still, current data on Cr levels in overweight and obese people are limited. Lima et al. [33], however, observed Cr deficiency in 87.7% of all obese people, while Tascilar et al. [34] and Azab et al. [35] found no variations in Cr levels between normal weight and obese children. In turn, according to a study by Son et al. [36], there is a relationship between increased chromium (Cr) levels in toenails and the incidence of metabolic syndrome.

Iron (Fe) is required in humans for erythropoiesis, the creation of myoglobin for oxygen delivery to muscle cells, host defense, DNA replication and repair, a variety of metabolic enzymes and energy production in mitochondria. In contrast, excess iron is toxic, as iron (II) produces highly reactive hydroxyl radicals through the Fenton reaction, causing damage to biological components such as lipids, DNA and proteins, resulting in tissue damage [37]. Obesity and iron deficiency are widespread global health problems that have an impact on billions of people [38,39]. While being overweight and obese are major risk factors for many chronic diseases [40], iron deficiency, also known as hypoferremia, is the most common single micronutrient deficiency [41]. There is growing evidence to support the existence of a relationship between overweight and obesity and iron deficiency [42]. According to an observational study in a group of 619 women, iron deficiency was observed in 23.5% of all women with normal body weight, 41.9% of all overweight respondents and 45.6% of all obese respondents [43]. Other observational studies involving 500 adults (*n* = 500) revealed that the average serum iron levels in men with normal body weight, overweight, class 1 obesity and class 2 obesity were 72.6 µg/dL, 64.2 µg/dL, 59.1 µg/dL and 54.7 µg/dL, respectively, while in women it was 61.2 µg/dL, 52.9 µg/dL, 44.8 µg/dL and 39.6 µg/dL, respectively. Hepcidin levels and chronic low-grade inflammation are thought to play a significant role in the link between overweight, obesity and hypoferremia [44].

While many researchers have assessed the correlation between concentrations of heavy metals and obesity, not much is known about the impact of heavy metal concentrations on the incidence of obesity among patients following myocardial infarction (MI). Due to the possible overlap in the biological mechanisms between heavy metal toxicity and obesity, as well as the impact of both on cardiovascular risk, obesity may intensify the harmful effects of heavy metals on the risk of cardiovascular incidents. This emphasises the significance of identification of high-risk populations for focused interventions, as well as the need for additional research into possible interactions between these two categories of risk factors. The study aimed at assessing concentrations of selected trace elements and heavy metals (Cu, Zn, Mn, Co and Fe) and ferritin, with anthropometric indices and physiological parameters associated with overweight and obesity in patients with and without a history of myocardial infarction.

## 2. Materials and Methods

### 2.1. Study Design and Participants

Between August and September 2017, 146 respondents participated in the cross-sectional study divided into the study group (SG) and control group (CG). The SG was made up of post-MI patients undergoing early cardiac rehabilitation (up to 14th day after hospital discharge from complete revascularisation), which included physical therapy, dietary and pharmacological therapy. The research material was collected at the treatment facility “Uzdrowisko Nałęczów” S.A. in Nałęczów and the Railway Health Resort Hospital in Nałęczów in the eastern Poland. The study group consisted of patients staying at rehabilitation camps held at 21 or 28-day intervals. All of the SG-eligible patients had undergone their first percutaneous coronary intervention (PCI) following their first MI. The SG inclusion criteria included: (1) age between 40 and 65 years, (2) first-ever MI incident and (3) written informed consent to participate in the study. The exclusion criteria were as follows: (1) diagnosed renal failure, (2) diagnosed neoplastic disease, (3) diagnosed respiratory disease, (4) diagnosed rheumatic disease, (5) age below 40 years and above 65 years, (6) taking dietary supplements or vitamin complexes that have impact on heavy metal concentrations, and (7) not giving informed written consent to take part in the study.

The CG-eligible patients were those that had regular medical examinations with an occupational medicine physician at the Provincial Occupational Medicine Centre, Prevention and Treatment Centre in Lublin in the eastern Poland. The inclusion criteria were as follows: (1) age between 40 and 65 years, (2) no cardiovascular incident, low 10-year risk of cardiovascular incident (Systematic Coronary Risk Evolution < 5), (3) no diagnosed chronic diseases such as: renal failure, neoplastic disease, rheumatic disease, pulmonary disease, hypertension, diabetes mellitus and pre-diabetes, hypercholesterolaemia, (4) no history of cardiovascular complaints indicating atherosclerotic cardiovascular disease (CVC), (5) no taking of trace element-containing dietary supplements. The exclusion criteria included: (1) active infection, (2) taking dietary supplements or vitamin complexes that have impact on heavy metal concentrations, and (3) not giving informed written consent to take part in the study.

To determine whether the respondent fulfilled the inclusion criteria, specially trained nurses performed a thorough interview in the SG and CG during the first visit. The respondents that were qualified for further testing were then examined and subjected to additional tests (anthropometric measurements). They were also instructed that the next morning before breakfast, they would have their blood taken for laboratory tests, and as a result, they were asked to respect the 12-h fast and not to eat or drink anything other than water.

### 2.2. Data Collection

#### 2.2.1. Anthropometric Measurements and Determination of Anthropometric Indices Associated with Overweight and Obesity

Anthropometric measurements of body weight and height were obtained for all of the respondents. A platform scale was used to measure body weight (without shoes or outer clothes) to the nearest 0.1 kg, while an altimeter was used to measure height to the nearest 0.1 cm. In the subsequent stage, BMI was assessed for each individual (defined as body weight in kilograms (kg) divided by height in meters squared (kg/m^2^)). Waist circumference (WC) was measured at the midpoint between the lowest rib margin and the top of the hip bone, while hip circumference (HC) was taken at the height of the greater trochanter of the femur. These measurements were taken with a rigid measuring tape. During both measurements, the individual exhaled and spread his or her legs 25 to 30 cm apart to distribute body weight. The measurements were obtained while a person was standing. Subsequently, the waist-to-hip ratio (WHR) and waist-to-height ratio (WHtR) were determined. Based on anthropometric measurements, all of the respondents had their Body Adiposity Index (BAI) (BAI = [HC (cm)/height (m)^1.5^] − 18) calculated [45]. Body fat percentage (FM%) was calculated using the Electrical Bioimpedance by means of a body fat analyser (OMRON Model BF306, Osaka, Japan) following the manufacturer’s approved algorithm.

#### 2.2.2. Blood Sample, Metal Biomarker Levels and Ferritin Level

Blood samples were drawn in a fasting state from the ulnar vein between 7:00 and 9:00 a.m. the next morning after an overnight rest. The samples were then collected in a tube containing a clotting activator and a separator (granules), which were transferred to the laboratory in less than one hour. The blood samples were kept at 4°C in an upright position with no access to light until they were transported to the laboratory. The plasma was centrifuged for 10 min at 3000 rpm. Following centrifugation, the serum was immediately transferred into Eppendorf tubes, frozen at −80 °C, and kept until ferritin and heavy metal concentrations (Cu, Zn, Mn, Co, Cr, and Fe) were measured.

Merck (Darmstadt, Germany) supplied Suprapur nitric acid (65% HNO_3_). Ultrapure water (Milli-Q, Millipore, Raleigh, NC, USA, resistivity 18.2 MΩ·cm) was used to prepare 1% HNO_3_ and dilute the samples. Additionally, Agilent Technologies (Santa Clara, CA, USA) provided certified single-element standard solutions of Cu, Zn, Mn, Co, Cr, and Fe, each at a concentration of 1000 mg/L and purity grade 99.99%. The approved reference material ClinChek^®^ Plasma Control for Trace Elements, Level I (München, Germany) was created according to the manufacturer’s specifications.

After collecting 0.2 mL of human plasma samples, 2 mL of 65% HNO_3_ (Merck, Darmstadt, Germany) were added to the organic matter for digestion. Wet mineralization was carried out utilising a Mars 6 microwave mineralisation system (CEM, Matthews, NC, USA). Following the addition of 2 mL of 65% HNO_3_, the samples were moved to Teflon containers and digested at 180 °C. Subsequently, the samples were diluted with 5 mL of ultrapure water and tested to determine trace element concentrations.

The Cu, Zn, Mn, Co, Cr, and Fe concentrations in the diluted samples were ascertained through inductively coupled plasma mass spectrometry (ICP-MS) (820-MS, Varian, Australia). Samples containing low concentrations of metal ions were pre-concentrated by lyophilization and subsequently diluted to the appropriate volume prior to analysis. The ICP-MS analysis was performed with the following parameters: plasma gas flow 18 L/min, nebuliser gas flow 1 L/min, frequency power 1.37 kW and auxiliary gas flow 1.70 L/min. To produce the calibration curve (corresponding to trace element concentrations in human plasma), the approved single-element standards were combined and diluted in 1% HNO_3_. The plasma and quality control samples were tested three times, and the average values were used to determine the final results. The analyses were carried out using an ISO 17025- accredited [46]. Method. Validation parameters of the ICP-MS procedure, including the limits of detection (LOD), limits of quantification (LOQ), precision, recovery, and expanded uncertainty, are shown in Table 1.

Serum ferritin level was determined using Enzyme-Linked Immunosorbent Assay (ELISA) technique and the Human Ferritin ELISA kit was obtained from ThermoFisher Scientific (Waltham, MA, USA). In brief, all reagents and serum samples were equilibrated to room temperature before the assay. Next, 100 μL of serum samples or standards (for the standard curve) was added to the wells precoated with anti-ferritin antibody. The 96-well plate was sealed with plate sealer and incubated at 37 °C for 90 min. After incubation, the following solutions were sequentially added to each well: Biotinylated Detection Antibody Working Solution, HRP Conjugate Working Solution and Substarate Reagent. After the final incubation (15 min at 37 °C), 50 μL of Stop Solution was added and the absorbance was determined by means of a microplate reader (Epoch, BioTek Instruments, Inc., Winooski, VT, USA) at 450 nm within next 10 min. The ferritin concentration in each sample was determined using the standard curve.

#### 2.2.3. Socio-Demographic Variables

Details such as age, gender, place of residence, education and smoking status were collected by means of a standardised survey questionnaire. Cigarette smokers were respondents that had smoked one or more cigarettes within the previous month.

### 2.3. Ethical Considerations

The research project was approved by the Bioethics Committee of the Medical University of Lublin (KE-0254/197/2017) and was carried out in line with the Declaration of Helsinki. All respondents were given an explanation of the study’s purpose before being requested to submit written consent to take part in the survey.

### 2.4. Statistical Analysis

Continuous variables were presented as medians with first quartile (Q1) and third quartile (Q3), or means with standard deviation (SD) if regularly distributed. The Kolmogorov–Smirnov test was employed to determine if the data followed a normal distribution. The differences in numerical variables between SG and CG groups were compared by means of *t*-test or by using Mann–Whitney U test. Categorical variables were presented as counts and percentages and were compared through Pearson’s chi-squared test. Spearman’s rank correlation was applied to investigate the relationships between the level of trace elements and heavy metals, ferritin and anthropometric measurements. Furthermore, potential confounding (age, sex, place of residence, education level and smoking status) effects were taken into account through the application of multiple regression and linear regression. The dependent variables were anthropometric measurements. The results are presented as standardized coefficients (b) with standard error (SE). Statistical analyses were conducted using IBM Corp. Released 2017. IBM SPSS Statistics for Windows, Version 25.0. Armonk, NY, USA: IBM Corp. Statistical and Statistica 13 PL (StatSoft, Tulsa, OK, USA). A *p* < 0.05 was considered statistically significant.

## 3. Results

### 3.1. Baseline Characteristics of the Participants

Table 2 presents the characteristics of the study group. The mean age in the SG was 57.38 ± 4.72 years and in the CG, it was 53.67 ± 6.32 years. No significant differences between the study groups by gender were identified. In terms of anthropometric indices linked to overweight and obesity, the SG had substantially higher values of BMI, WC, WHR, and WHtR than the CG (*p* < 0.05).

With regard to serum concentrations of trace elements and heavy metals, the post-MI group had considerably higher levels of Cu, Zn, Mn, Co, Fe and Zn/Fe than the CG-eligible respondents (*p* < 0.05). In addition, ferritin levels were significantly higher in the SG (*p* = 0.007).

### 3.2. Correlation Between Heavy Metal and Ferritin Concentrations in Blood Serum and Anthropometric Indices Associated with Overweight and Obesity

Table 3 shows the correlation between heavy metal and ferritin concentrations in serum and anthropometric indices and physiological parameters connected with overweight and obesity. In the SG, Cr was positively correlated with BAI, whereas Zn, the Zn/Cu ratio, and ferritin were each negatively correlated with body fat percentage.

In contrast, in the CG, there was a positive correlation between Zn concentration, and WHtR and between ferritin level, and BMI, WC, WHR and WHtR. Additionally, a negative correlation was found between Mn concentration, and WHR and ferritin level, and BAI and FM%.

Table 4 shows the results of multiple linear regression. After adjusting for age, sex, place of residence, education level and smoking status, a positive association was discovered between Cr and BAI, and a negative association between FM% and Zn in the SG. Additionally, there was a tendency evident between FM% and Zn/Cu. In the CG, the statistical significance of all associations that were significant in the univariable analysis were lost after accounting for potential confounding factors.

## 4. Discussion

In recent years, the possible negative impact of environmental exposure to heavy metals on human health has raised concerns for public health worldwide. Global urbanisation and industrialisation have led to an increase in the risk of human exposure to heavy metals. In this cross-sectional study, we assessed the correlation between concentrations of selected trace elements and heavy metals (Cu, Zn, Mn, Co, Cr and Fe) and ferritin in blood serum, and anthropometric indices associated with overweight and obesity in patients with and without a history of myocardial infarction. The outcome of this work was evidence that both study groups’ anthropometric and physiological parameters associated with obesity showed positive correlations with ferritin and certain heavy metals.

In this study, we discovered a positive correlation between the post-MI group’s body fatness indices and Cr concentration. The exact mechanism linking Cr to excess body fat is unknown, but it is thought to be related to carbohydrate and lipid metabolism, where it may play a significant role in supporting insulin action in controlling blood glucose levels and thus influencing body weight [47]. This is of key importance in lowering cardiometabolic risk factors, particularly in individuals who have experienced a cardiovascular event. Glucose tolerance factor (GTF) is a key component of glucose metabolism. It is a substance that regulates blood glucose levels by facilitating glucose transfer into cells. GFT is a complex that includes chromium (III), nicotinic acid and the amino acids, i.e., glutamic acid, glycine and cysteine. In addition, chromium (III) is a component of chromodulin, a low-molecular-weight Cr-binding molecule that enhances insulin receptor activity by phosphorylation [48]. Chen et al. [37,49] found that Cr increases insulin-dependent translocation of glucose transporters from the cytoplasm to the cell membrane, hence commencing active glucose transport into the cells. Chromium (Cr) regulates blood glucose levels as well as lipid metabolism. Research has shown that supplementing with high levels of Cr decreased total cholesterol, LDL cholesterol (LDL-C), unsaturated fatty acids and triglycerides in blood serum, while increasing HDL cholesterol and beta-oxidation [50]. Evidence suggests that Cr supplementation lowers cardiovascular risk and insulin resistance [51]. Lewicki et al. [52], for instance, concluded that Cr supplementation reduces the risk of atherosclerosis, heart attack and cholesterol levels in obese people.

Chromium is widely promoted as a weight loss supplement because of its ability to regulate eating habits and cravings, suppress appetite, induce thermogenesis, increase resting energy expenditure and improve insulin sensitivity [53]. Animal studies have shown that chromium may reduce body weight in obese rats [54], but systematic reviews have demonstrated that chromium supplementation is ineffective in weight loss in overweight and obese adults due to low evidence quality [55]. Tsang et al. [53] have conducted a meta-analysis of 19 randomised controlled trials to determine the efficacy of oral chromium supplementation in overweight or obese individuals. The meta-analysis found that chromium supplementation in the form of chromium picolinate, chromium nicotinate, or chromium-enriched yeast resulted in significant decreases in total body weight, BMI, and body fat percentage in overweight and obese people. Nevertheless, following chromium supplementation, the meta-analysis results did not demonstrate a decrease in WC and WHR, i.e., the visceral obesity indices most strongly associated with cardiometabolic risk. Similar conclusions were drawn in the meta-analysis by Onakpoya et al. [55]. They obtained results that are akin to those mentioned above. Still, we second the authors of the two meta-analyses referred to above in suggesting that further research is needed to determine how chromium levels affect body weight. This should be done by comparing such levels to a variety of anthropometric and physiological parameters related to overweight or obesity.

It is widely recognised that zinc supplementation helps treat obesity by reducing weight loss, lowering inflammatory markers, and decreasing insulin resistance, however, the mechanisms behind the relationship between serum Zn level and overweight and obesity are not fully understood. According to published research, three mechanisms may explain the relationship between Zn level and excess body fat.

In terms of the first mechanism, excess body fat is associated with chronic inflammation and oxidative stress that has an impact on glucocorticoid synthesis and, as a result, promotes the expression of genes encoding metallothionein and zinc transporters. These proteins enhance Zn absorption by adipocytes, thereby reducing its concentration in blood serum [17,18,56]. Additionally, adipose tissue releases a variety of cytokines, including interleukin-6 (IL-6), interleukin-8 (IL-8), tumour necrosis factor alpha (TNF-α) and leptin [4,57]. Pro-inflammatory adipocytokines can trigger the expression of Zn transporters, which may also contribute to a decrease in its concentration in blood serum [17,58,59].

The second mechanism involves oxidative stress, which is related to excess body fat. Zinc (Zn) is a component of numerous antioxidant enzymes, including superoxide dismutase (SOD), but it also contributes to lipid oxidation [4,60]. Zinc (Zn) can lower TNF-α levels by affecting zinc-alpha-2-glycoprotein (ZAG), which is involved in TNF-α transcription [58]. Zinc deficiency may increase TNF-α levels in the blood, resulting in the reactive oxygen species (ROS) release in tissues and increased oxidative stress [5]. This directly contributes to obesity-related complications and cardiometabolic risk [58].

The third potential mechanism is related to serum leptin concentration, i.e., adipokine associated with satiety, through the influence of ZAG on its concentration [36,61,62,63]. Elevated blood leptin levels lead to leptin resistance in obese people through disturbances at many levels of the leptin signalling. These impairments include decreased hormone accessibility to its receptor as a result of altered post-receptor signal transduction or altered receptor expression [64,65,66]. Adipose tissue produces more leptin to counteract the consequences of leptin resistance, which leads to obesity and leptin resistance, resulting in a vicious cycle [67,68]. In addition, leptin plays a critical role in increasing the development of inflammatory cytokines (for instance, IL-6, IL-8 and TNF-α) [69], the levels of which contribute to inflammation in individuals with excess body fat and may contribute to obesity-related inflammation [70].

In a case–control study, Mota Martins et al. [18] discovered a negative correlation between excess body weight and Zn body levels in erythrocytes and plasma. Similarly, in our study, Zn concentration and percentage body fat in post-MI patients demonstrated a negative correlation. However, in patients without myocardial infarction, a positive correlation was found between Zn concentration and WHtR, which is in contrast to the previous studies. This may result from the methodology of the anthropometric measurements or from medications taken that may affect serum Zn levels. In addition, Zn interferes with the metabolism of other trace elements, such as calcium, phosphorus and copper, among others [71]. The levels of zinc and copper are strictly regulated by compensatory mechanisms that act to balance their concentrations. Low Zn levels and high Cu levels can cause oxidative stress and impair many antioxidant enzymes [72]. In terms of Zn and Cu’s antagonistic relationship, each is absorbed through a competitive mechanism, which means that an excess of one causes a deficiency of the other. According to Kärberg et al. [73], the Zn/Cu ratio is correlated with diet and plays a major role in the pathogenesis of metabolic diseases. The authors’ study showed a negative correlation between the percentage of body fat in post-MI patients and the Zn/Cu ratio.

Serum ferritin reflects body iron storages and plays a major role in maintaining iron balance. Indeed, some studies have shown reduced iron concentrations in overweight and obese individuals [45]. The primary mechanism connecting excess body fat and iron deficiency is thought to be related to the low-grade inflammation that is present in obese individuals [74]. Several studies with contradictory results found a link between ferritin levels and obesity, but correlation between high ferritin levels and obesity is not readily apparent [75]. However, other studies have revealed that obese individuals have similar or even lower levels of ferritin as compared to normal weight people [76]. In the authors’ study, ferritin levels were positively associated with BMI, WC, WHR and WHtR in patients without a cardiovascular incident, and negatively associated with percentage body fat in both groups and BAI in those without a cardiovascular incident in univariable models. However, in multivariable models, after taking into account confounding variables, the relationships lost their significance. A similar positive association between ferritin levels and BMI was observed by Hitha et al. [77]. Moreover, Ding et al. [78] determined that ferritin levels were associated with insulin resistance, visceral adiposity index (VAI) and lipid accumulation product (LAP). Serum ferritin is a widely recognised and effective inflammatory disease marker in both acute and chronic illnesses [79]. According to previous research, abdominal obesity is more significantly related to inflammatory markers than BMI or total body fat [80].

The positive correlation between Mn concentration and WHtR in patients without a cardiovascular incident is consistent with data regarding lower dietary Mn intake in obese individuals [81], which also supports the findings made by Skalnaya et al. [82] concerning the lower level of this element in the hair of obese people. In experimental studies on animals, adequate Mn supplementation significantly reduced abdominal fat content in broilers.

Given current state of knowledge, explaining the link between Mn level and excess body fat is difficult. However, it is known that Mn is a vital component of metalloenzymes and is involved in the metabolism of carbohydrates, proteins and lipids [24]. Manganese (Mn) supplements may help reduce abdominal fat accumulation by lowering fatty acid synthesis and malate dehydrogenase activity in the liver [83] and glycerol in adipose tissue [84], as well as total cholesterol and LDL-C levels, as Mn may be a crucial element of lipoprotein structure [85]. Manganese (Mn) is one of the essential components of manganese superoxide dismutase (MnSOD), which may be associated with the development of atherosclerosis by reducing oxLDL-induced apoptosis of macrophages [86,87]. In addition, Mn provides protection against vascular endothelial dysfunction [88] and inhibits LDL oxidation by vascular endothelial cells [89]. The association between reduced MnSOD activity and atherogenesis suggests that analysis of Mn level in the blood vessel wall matrix may be a diagnostic method for identifying early atherosclerotic changes [90].

This study has a few significant limitations. Firstly, since our study is cross-sectional, it does not demonstrate a cause-and-effect relationships or temporal relationships between plasma metal concentrations and physiological and anthropometric indices related to obesity and overweight. Secondly, as every metal has a unique distribution throughout the organs and circulatory system, there is currently no one accepted method for metal determination. It is likely that determination of their plasma concentrations does not accurately represent the overall concentrations of particular metals in the human body. However, the analysis concluded by Yuan et al. [25] of the correlation of concentrations of certain heavy metals in plasma, whole blood and urine samples suggests comparable levels of concentrations of the metals we studied in plasma, whole blood and urine. Thirdly, our study did not consider lifestyle factors, such as diet or other variables that could have altered trace element and heavy metal levels, such as medication. However, one of the inclusion criteria for the study group was that respondents did not take any dietary supplements or vitamin complexes that could affect the levels of the elements under study. Future study should consider lifestyle aspects, including respondents’ diet and regular medications. Fourthly, the possible consequence of a cardiovascular event and its treatment in the study group may be related to inflammation and may affect ferritin and trace element levels. Fifthly and lastly, as a result of the cardiovascular event and hospitalisation, the respondents in the study group underwent tests to diagnose comorbidities, such as metabolic disorders, hypertension, diabetes, and so on. In turn, respondents in the control group may have been unaware of the existence of such diseases, and because they had no symptoms and had not been diagnosed, they claimed to have no comorbidities. In addition, the myocardial infarction experienced by respondents in the study group could have an impact on the trace element and heavy metal levels under study. As a result, these discrepancies may make comparisons between the two groups difficult or limit the possibility of generalisation.

## 5. Conclusions

In summary, this study has shown an association between several serum concentrations of trace elements and heavy metals, and anthropometric and physiological indices related to overweight and obesity. Even though the associations between latter and the former are not entirely clear, this study has significant implications for public health, particularly in light of the growing environmental exposure to heavy metals. Our study results may be useful in developing preventive strategies to minimise overweight and obesity, both in the primary and secondary prevention of cardiometabolic risk. Further research is required to fully determine the association between heavy metals and the prevalence of overweight and obesity.

## Figures and Tables

**Table 1:** Results of method validation for ICP-MS determination of trace elements in human plasma.

Element	LOD [mg/kg]	LOQ [mg/kg]	Precision [%]	Recovery [%]	Expanded Uncertainty [%]
Fe	4.3	8.6	8.6	96	9
Cu	1.3	2.6	2.3	99	20
Zn	0.4	0.9	5.1	106	14
Mn	1.1	2.2	9,2	97	7
Co	0.012	0.024	7.12	101	11
Cr	0.015	0.030	3.63	97	16

LOD: limits of detection; LOQ: limits of quantification.

**Table 2:** Characteristics of the analysed study group.

Variables	Study Group (*n* = 74)	Control Group (*n* = 72)	*p*
Demographic data:			
Age [years] ^b^	57.38 ± 4.72	53.67 ± 6.32	<0.001
Gender (male) ^a^	54 (73.0)	44 (61.1)	0.13
Place of residence (city) ^a^	31 (41.9)	49 (68.1)	0.001
University education ^a^	14 (18.9)	43 (59.7)	<0.001
Current smoking ^a^	22 (29.73)	7 (9.72)	0.002
Anthropometric variables:			
BMI [kg/m^2^] ^b^	28.78 ± 4.97	26.58 ± 4.12	0.004
WC [cm] ^b^	103.54 ± 12.78	93.12 ± 12.85	<0.001
HC [cm] ^b^	104.95 ± 10.79	103.32 ± 7.77	0.3
WHR ^b^	0.99 ± 0.08	0.9 ± 0.09	<0.001
WHtR ^b^	0.6 ± 0.07	0.54 ± 0.07	<0.001
BAI ^b^	28.57 ± 6.38	28.25 ± 4.68	0.72
FM% ^b^	30.83 ± 7.49	30.2 ± 7.05	0.6
Selected trace elements, heavy metals and ferritin in blood serum:			
Cu (10^−6^) µg/mL ^c^	0.97 (0.83–1.13)	0.72 (0.62–0.87)	<0.001
Zn (10^−6^) µg/mL ^c^	0.45 (0.40–0.55)	0.36 (0.33–0.42)	<0.001
Mn (10^−9^) ng/mL ^c^	2.42 (1.17–4.55)	0.27 (0.12–0.54)	<0.001
Co (10^−9^) ng/mL ^c^	0.33 (0.26–0.43)	0.17 (0.12–0.26)	<0.001
Cr (10^−9^) ng/mL ^c^	130.89 (109.29–173.86)	132.26 (115.26–150.41)	0.69
Fe µg/mL ^c^	0.78 (0.66–1.02)	0.51 (0.38–0.66)	<0.001
Zn/Fe ^c^	0.61 (0.38–0.81)	0.722 (0.54–1.22)	0.005
Zn/Cu ^c^	0.47 (0.38–0.58)	0.48 (0.38–0.67)	0.272
Ferritin [ng/mL] ^c^	148.18 (61.11–215.35)	88.25 (27.68–163.58)	0.007

Date is presented as: ^a^ n (%); ^b^ mean ± SD; ^c^ median (Q1–Q3). BMI: body mass index; WC: waist circumference; HC: hip circumference; WHR: waist to hip ratio; WHtR: waist-to-height ratio; BAI: body adiposity index; FM%: body fat percentage.

**Table 3:** Correlation between selected trace elements, heavy metal and ferritin concentrations in blood serum and anthropometric indices related to overweight and obesity.

		Cu	Zn	Mn	Co	Cr	Fe	Zn/Fe	Zn/Cu	Ferritin
Study group:										
BMI	r	−0.007	−0.156	−0.009	−0.056	0.15	−0.087	−0.036	−0.117	0.108
	*p*	0.951	0.185	0.937	0.637	0.202	0.46	0.759	0.321	0.34
WC	r	−0.014	−0.09	0.063	0.029	0.156	−0.016	−0.068	−0.049	0.152
	*p*	0.909	0.444	0.599	0.807	0.186	0.893	0.562	0.676	0.179
HC	r	−0.049	−0.053	0.038	0.03	0.216	−0.152	0.064	−0.007	0.092
	*p*	0.68	0.656	0.749	0.749	0.065	0.195	0.589	0.954	0.415
WHR	r	−0.083	−0.054	0.001	0.043	−0.043	0.174	0.058	0.212	−0.187
	*p*	0.487	0.65	0.995	0.72	0.718	0.137	0.626	0.059	0.111
WHtR	r	−0.02	−0.138	−0.069	−0.099	0.21	−0.067	−0.056	−0.085	0.03
	*p*	0.864	0.241	0.567	0.405	0.073	0.572	0.634	0.472	0.789
BAI	r	0.009	−0.104	−0.129	−0.153	0.265	−0.207	0.071	−0.109	−0.091
	*p*	0.937	0.379	0.28	0.197	0.023	0.077	0.547	0.357	0.423
FM%	r	0.158	−0.252	−0.175	−0.15	0.073	−0.119	−0.092	−0.354	−0.285
	*p*	0.181	0.03	0.142	0.205	0.538	0.315	0.436	0.002	0.011
Control group:										
BMI	r	−0119	0.129	−0.05	−0.143	−0.034	0.013	0.068	0.146	0.392
	*p*	0.321	0.282	0.679	0.23	0.777	0.917	0.572	0.226	<0.001
WC	r	−0.196	0.207	−0.19	−0.123	0.132	0.087	0.061	0.22	0.486
	*p*	0.098	0.083	0.111	0.303	0.269	0.467	0.616	0.066	<0.001
HC	r	−0.079	0.101	0.027	0.011	0.026	0.112	−0.001	0.155	0.155
	*p*	0.51	0.402	0.824	0.93	0.829	0.349	0.993	0.171	0.171
WHR	r	−0.191	0.191	−0.239	−0.211	0.199	0.06	0.041	0.195	0.549
	*p*	0.109	0.111	0.043	0.075	0.094	0.616	0.732	0.104	<0.001
WHtR	r	−0.124	0.235	−0.12	−0.031	0.142	0.021	0.114	0.223	0.308
	*p*	0.3	0.048	0.317	0.795	0.234	0.858	0.342	0.062	0.006
BAI	r	0.111	−0.039	0.112	0.19	0.007	−0.098	0.056	−0.024	−0.357
	*p*	0.351	0.748	0.349	0.109	0.953	0.412	0.644	0.842	0.001
FM%	r	0.075	−0.048	0.119	0.227	−0.21	−0.148	0.124	−0.004	−0.483
	*p*	0.53	0.693	0.319	0.055	0.077	0.215	0.301	0.975	<0.001

BMI: body mass index; WC: waist circumference; HC: hip circumference; WHR: waist to hip ratio; WHtR: waist-to-height ratio; BAI: body adiposity index; FM%: body fat percentage; r: correlation coefficient.

**Table 4:** Multivariable association between selected trace elements, heavy metal and ferritin concentrations in blood serum and anthropometric indices related to overweight and obesity.

		Cu	Zn	Mn	Co	Cr	Fe	Zn/Fe	Zn/Cu	Ferritin
Study group:										
BMI	b^A^ (SE)	−0.1 (0.126)	−0.216 (0.118)	−0.075 (0.122)	−0.134 (0.121)	0.118 (0.119)	−0.081 (0.118)	−0.084 (0.12)	−0.15 (0.125)	0.103 (0.122)
	*p*	0.429	0.072	0.542	0.274	0.325	0.493	0.487	0.233	0.403
WC	b^A^ (SE)	−0.068 (0.12)	−0.2 (0.114)	−0.096 (0.116)	−0.104 (0.117)	0.153 (0.114)	−0.062 (0.113)	−0.092 (0.115)	−0.155 (0.12)	0.02 (0.119)
	*p*	0.574	0.083	0.411	0.377	0.183	0.584	0.427	0.2	0.864
HC	b^A^ (SE)	−0.095 (0.124)	−0.132 (0.118)	−0.027 (0.12)	−0.09 (0.12)	0.211 (0.115)	−0.085 (0.116)	−0.003 (0.119)	−0.081 (0.124)	0.056 (0.119)
	*p*	0.446	0.268	0.825	0.455	0.071	0.465	0.982	0.513	0.638
WHR	b^A^ (SE)	0.017 (0.103)	−0.149 (0.097)	−0.09 (0.099)	−0.037 (0.099)	−0.023 (0.098)	0.01 (0.096)	−0.131 (0.097)	−0.143 (0.101)	−0.041 (0.1)
	*p*	0.871	0.13	0.362	0.713	0.816	0.92	0.183	0.161	0.683
WHtR	b^A^ (SE)	−0.096 (0.124)	−0.194 (0.117)	−0.148 (0.119)	−0.141 (0.119)	0.182 (0.116)	−0.074 (0.116)	−0.053 (0.119)	−0.128 (0.123)	0.04 (0.12)
	*p*	0.44	0.102	0.215	0.24	0.121	0.525	0.655	0.301	0.74
BAI	b^A^ (SE)	−0.104 (0.106)	−0.097 (0.1)	−0.089 (0.101)	−0.123 (0.1)	0.201 (0.097)	−0.085 (0.098)	0.051 (0.1)	−0.037 (0.105)	0.071 (0.099)
	*p*	0.328	0.338	0.381	0.224	0.042	0.392	0.616	0.727	0.473
FM	b^A^ (SE)	−0.078 (0.096)	−0.224 (0.086)	−0.047 (0.09)	−0.074 (0.091)	0.031 (0.09)	−0.033 (0.089)	−0.047 (0.09)	−0.166 (0.092)	0.054 (0.088)
	*p*	0.419	0.012	0.607	0.418	0.733	0.709	0.604	0.076	0.54
Control group:										
BMI	b^A^ (SE)	−0.094 (0.119)	0.018 (0.121)	−0.023 (0.119)	−0.058 (0.125)	−0.11 (0.125)	−0.048 (0.119)	−0.01 (0.12)	0.054 (0.121)	0.16 (0.142)
	*p*	0.431	0.884	0.844	0.643	0.384	0.69	0.933	0.654	0.264
WC	b^A^ (SE)	−0.089 (0.108)	0.097 (0.109)	−0.072 (0.107)	0.047 (0.113)	−0.015 (0.114)	0.059 (0.108)	0.077 (0.108)	0.116 (0.108)	0.18 (0.129)
	*p*	0.413	0.378	0.503	0.682	0.895	0.585	0.481	0.289	0.168
HC	b^A^ (SE)	−0.092 (0.125)	0.165 (0.125)	−0.076 (0.124)	0.004 (0.131)	0.005 (0.132)	0.003 (0.125)	0.09 (0.125)	0.201 (0.124)	0.228 (0.148)
	*p*	0.465	0.191	0.544	0.977	0.973	0.984	0.477	0.111	0.13
WHR	b^A^ (SE)	−0.051 (0.097)	0.005 (0.098)	−0.037 (0.096)	0.058 (0.101)	−0.022 (0.102)	0.086 (0.096)	0.029 (0.098)	0.005 (0.098)	0.062 (0.115)
	*p*	0.604	0.962	0.702	0.569	0.832	0.377	0.768	0.956	0.594
WHtR	b^A^ (SE)	−0.103 (0.12)	0.063 (0.122)	−0.054 (0.12)	0.072 (0.126)	0.03 (0.127)	0.052 (0.12)	0.056 (0.121)	0.078 (0.122)	0.194 (0.144)
	*p*	0.395	0.605	0.653	0.57	0.813	0.666	0.645	0.526	0.181
BAI	b^A^ (SE)	−0.075 (0.103)	0.057 (0.104)	−0.016 (0.102)	0.05 (0.108)	0.1 (0.108)	−0.029 (0.103)	0.027 (0.104)	0.068 (0.104)	0.168 (0.12)
	*p*	0.471	0.59	0.878	0.642	0.356	0.778	0.794	0.519	0.166
FM%	b^A^ (SE)	−0.127 (0.096)	0.077 (0.098)	0.064 (0.096)	−0.067 (0.101)	−0.051 (0.102)	−0.002 (0.097)	0.045 (0.097)	0.104 (0.098)	0.034 (0.115)
	*p*	0.188	0.435	0.508	0.507	0.619	0.983	0.643	0.289	0.769

b—the standardized regression coefficient; SE—standard error, BMI: body mass index; WC: waist circumference; HC: hip circumference; WHR: waist to hip ratio; WHtR: waist-to-height ratio; BAI: body adiposity index; FM%: body fat percentage; ^A^—after adjustment for age, sex, place of residence, education level and smoking status.

## Data Availability

The data presented in this study are available upon request from the corresponding author due to privacy reasons.
